# Hypoxia-Induced Epithelial-To-Mesenchymal Transition Mediates Fibroblast Abnormalities via EKR Activation in Cutaneous Wound Healing

**DOI:** 10.3390/ijms20102546

**Published:** 2019-05-24

**Authors:** Jihee Kim, Bomi Kim, Soo Min Kim, Chae Eun Yang, Seung Yong Song, Won Jai Lee, Ju Hee Lee

**Affiliations:** 1 Department of Dermatology, Cutaneous Biology Research Institute, Yonsei University College of Medicine, Seoul 03722, Korea; mygirljihee@yuhs.ac (J.K.); BMKIM326@yuhs.ac (B.K.); soomin6306@yuhs.ac (S.M.K.); 2 Scar Laser and Plastic Surgery Center, Yonsei Cancer Hospital, Seoul 03722, Korea; pssysong@yuhs.ac (S.Y.S.); pswjlee@yuhs.ac (W.J.L.); 3 Department of Plastic and Reconstructive Surgery, Yonsei University Wonju College of Medicine, Wonju 26426, Korea; cheniya@yuhs.ac; 4 Department of Plastic and Reconstructive Surgery, Institute for Human Tissue Restoration, Yonsei University College of Medicine, Seoul 03722, Korea

**Keywords:** wound healing, ERK, hypoxia, fibroblast, scar

## Abstract

Previous studies described the involvement of extracellular signal-related kinase (ERK) in systemic fibrotic diseases, but the role of ERK in cutaneous scarring is unknown. Although hypoxia drives tissue fibrosis by activating hypoxia-inducible factor-1α (HIF-1α), the specific roles of hypoxia and associated ERK phosphorylation in abnormal fibroblast activity during cutaneous scarring are unclear. Here, we investigated whether pathologic myofibroblast-like keloid fibroblast activity is promoted by hypoxia-induced epithelial–mesenchymal transition mediated by ERK activation. ERK phosphorylation was significantly increased in keloid tissue and fibroblasts. Human dermal fibroblasts cultured under hypoxia (1% O_2_) expressed phosphorylated ERK and exhibited activation of p38 mitogen-activated protein kinase signaling. Hypoxic human dermal fibroblasts showed increased protein and mRNA levels of epithelial–mesenchymal transition markers. Furthermore, administration of an ERK inhibitor (SCH772984) reduced the hypoxia-induced elevation of collagen type I levels in human dermal fibroblasts. Therefore, ERK may be a promising therapeutic target in profibrogenic diseases.

## 1. Introduction

Wound healing normally progresses through coagulation, inflammation, proliferation, and remodeling phases. However, persistent tissue fibrosis due to abnormal dermal fibroblast activity and excessive synthesis of extracellular matrix (ECM) components is a common manifestation in cutaneous scarring [1,2]. Keloids and hypertrophic scars are types of excessive scarring that result from defects in the normal wound healing process. Keloid fibroblasts (KFs) acquire fibrogenic potential due to multiple factors, including an anomalous cellular response to mechanical strain, excessive release of growth factors and inflammatory cytokines, alterations in cellular apoptosis, and occurrence of the epithelial–mesenchymal transition (EMT) [2,3,4,5]. In contrast to normal fibroblasts, KFs gain a myofibroblast-like phenotype and possess tumor-like properties, and are able to proliferate and invade into surrounding tissue [6,7]. Despite much scientific and clinical interest, current treatment options are often ineffective and produce suboptimal results [1,8]. Thus, a better understanding of pathogenic wound healing would enable the development of more targeted treatment modalities for cutaneous scarring and fibrosis.

Previous reports demonstrate that KFs originate from the EMT of epithelial and endothelial cell components during wound healing [6,9], and keloid tissue shows increased expression of EMT markers [10]. Studies of progressive lung and kidney fibrosis show that inflammation and hypoxia promote the induction of EMT [11,12]. Hypoxia-inducible factor (HIF)-1α is a crucial mediator of cell metabolism, inflammation, and fibrosis under hypoxic conditions. Previous studies report that ECM accumulation in keloid tissue results from constant hypoxia and that HIF-1α is highly expressed in keloid tissue [13,14]. Although hypoxia-induced cutaneous fibrosis and the associated EMT process are known to be involved in keloid formation, the underlying molecular mechanisms are not well understood.

Transforming growth factor (TGF)-β1 regulates the healing response, and is implicated in tissue fibrosis and related diseases. Cellular stress activates extracellular signal-regulated kinase (ERK) [15], which is part of the mitogen-activated protein kinase (MAPK) signaling cascade. Among the MAPK pathways implicated in TGF-β1 responses [13], aberrant activation of the ERK pathway has been confirmed in systemic organ fibrosis, with ERK phosphorylation modulating fibroblast differentiation [16,17]. In tumor models, hypoxia stimulates ERK in different cell types [18], and hypoxia-induced ERK activation contributes to apoptosis resistance [19]. However, the link between hypoxia-induced ERK activation and ECM accumulation in cutaneous wound healing has not been investigated. 

We hypothesize that HIF-1α induces abnormal fibroblast activity via activation of the ERK signaling pathway, thereby leading to abnormal cutaneous scarring. Therefore, we elucidated the molecular mechanisms of ERK activation in cultured fibroblasts under hypoxia. 

## 2. Results

### 2.1. p-ERK and HIF-1α Levels Are Elevated in Keloid Tissue and Fibroblasts

**Figure 1:**
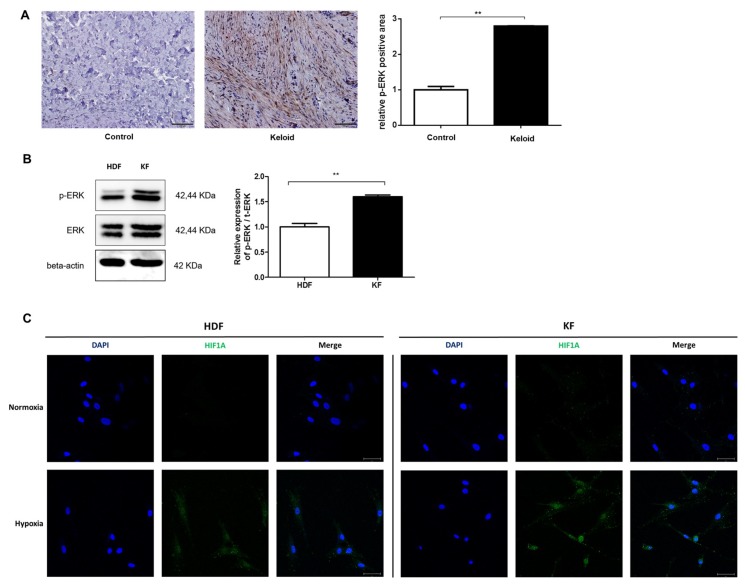
Phosphorylated extracellular signal-regulated kinase (p-ERK) levels in keloid tissue and fibroblasts. (**A**) Immunohistochemical analysis showed that the appearance of p-ERK was higher in keloid tissue than in adjacent normal tissue. Semi-quantitative analysis using Metamorph image analysis software revealed that the p-ERK levels were increased by 2.8-fold in keloid tissue compared with normal tissue. 100× magnification, scale bar = 200 µm. ** *p* < 0.01. (**B**) Western blot analysis showed that patient-derived keloid fibroblasts (KFs) had higher levels of *p*-ERK than human dermal fibroblasts (HDFs). ** *p* < 0.01. (**C**) Hypoxia-inducible factor-1α (HIF-1α) nuclear translocation in KFs was increased after exposure to 12 h of hypoxia; DAPI (blue), HIF-1α (green), scale bar = 50 µM.

We immunohistochemically examined keloid tissue from three patients, and found that phosphorylated ERK (p-ERK) levels were 2.8-fold higher in keloid tissue than in normal tissue (Figure 1a). We also measured basal p-ERK levels in different cell types using Western blot analysis, and found that patient-derived KFs had higher levels of p-ERK than those of normal human dermal fibroblasts (HDFs; Figure 1b). When we compared the expression of HIF-1α between KFs and HDFs after 12 h of hypoxia (1% O_2_), we found that KFs showed more nuclear translocation of HIF-1α compared with HDFs (Figure 1c).

### 2.2. Hypoxia Activates TGF-β Signaling and Induces EMT in HDFs

**Figure 2:**
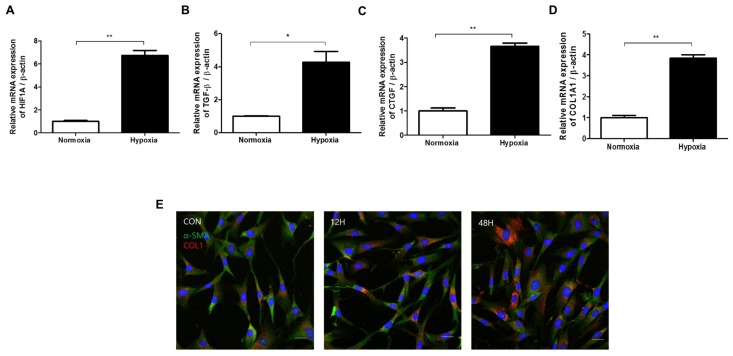
Effect of hypoxia on transcription factor-beta (TGF-β) signaling and epithelial–mesenchymal transition (EMT) marker expression in HDFs. Quantitative reverse transcription polymerase chain reaction (qRT-PCR) analyses of (**A**) HIF-1α, (**B**) TGF-β1, (**C**) connective tissue growth factor (CTGF), and (**D**) type I collagen mRNA 48 h after hypoxia exposure, as compared to a normoxia control group. (**E**) The amount of deposited collagen relative to total protein concentration was elevated in HDFs 0, 12, and 48 h after hypoxia exposure; DAPI (blue), α-SMA (green), type I collagen (red). Results are representative of three independent experiments, scale bar = 50 µM. Data are shown as mean ± SD. * *p* < 0.05, ** *p* < 0.01.

Next, we investigated the effect of hypoxia on HDFs. Quantitative reverse transcription polymerase chain reaction (qRT-PCR) analyses showed increased levels of connective tissue growth factor (CTGF), HIF-1α, TGF-β1, and type I collagen mRNA after 48 h of hypoxia, as compared with a normoxia control group (Figure 2a–d). TGF-β1 signaling is a pivotal fibrogenic factor that is responsible for EMT-like changes and causes abnormal ECM accumulation in keloid tissue [9]. CTGF is a fibrogenic cytokine expressed in several cell types, including fibroblasts and smooth muscle cells, and is induced by stimulation with TGF-β [20]. Immunofluorescence revealed that type I collagen deposition gradually increased for up to 48 h after hypoxia exposure (Figure 2e). These results suggest that HIF-1α triggered by hypoxia induces TGF-β1 signaling, leading to CTGF mRNA transcription and associated collagen deposition.

### 2.3. The ERK/MAPK Pathway Is Involved in Hypoxia-Induced EMT 

**Figure 3:**
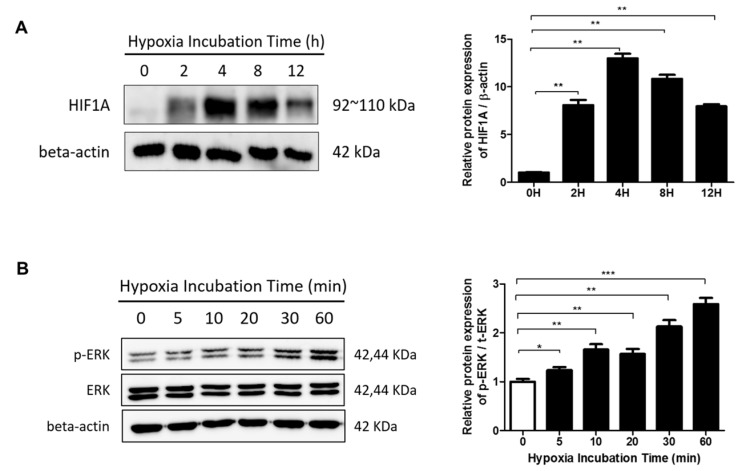
The effects of hypoxia on HIF-1α activation and extracellular signal-related kinase (ERK) phosphorylation in HDFs were analyzed by Western blot. (**A**) HIF-1α protein levels were increased in HDFs cultured under hypoxia for the indicated times. Data are shown as mean ± SD. ** *p* < 0.01 (**B**) Phosphorylation levels of ERK under hypoxia were assessed. Graphs show the optical density ratios between the bands representing the phosphorylated and total protein. Data are shown as mean ± SD. * *p* < 0.05, ** *p* < 0.01, *** *p* < 0.001.

To examine the effect of hypoxia on the expression of HIF-1α in HDFs, cells were cultured under hypoxic conditions for varying amounts of time. Hypoxia increased HIF-1α protein levels (Figure 3a), which peaked after 4 h and returned to basal levels after 24 h (data not shown). Previous reports show that MAPK/ERK signaling is activated by hypoxia and that HIF-1α is phosphorylated by an ERK-dependent mechanism [19,21]. To determine the downstream effector of HIF-1α activation, we examined the effect of hypoxia on ERK phosphorylation in HDFs within a time period of one hour using Western blot analysis. ERK phosphorylation gradually increased starting after exposure to hypoxia for 5 min (Figure 3b). 

**Figure 4:**
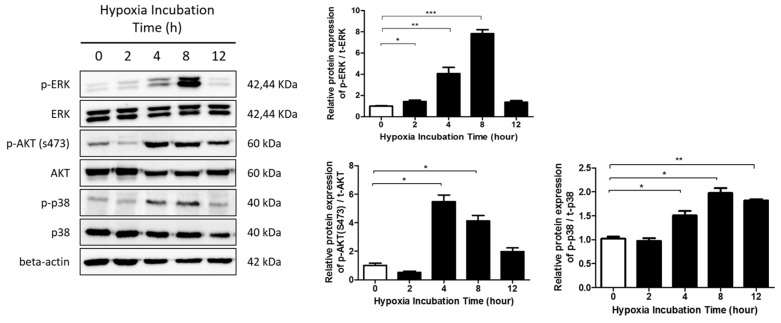
Involvement of the ERK/mitogen-activated protein kinase (MAPK) pathway in hypoxia-induced HIF-1α transcription. ERK phosphorylation was noted up to 8 h exposure to hypoxia. The AKT and p38 pathways were activated upon exposure to hypoxia for 12 h. Graphs show the optical density ratios between the bands representing the phosphorylated and total protein. Data are shown as mean ± SD. * *p* < 0.05, ** *p* < 0.01, *** *p* < 0.001.

We next examined the degree of ERK phosphorylation with prolonged exposure to hypoxia. We also evaluated the levels of activated phosphatidylinositol-4,5-bisphosphate 3-kinase (PI3K) and p38 in hypoxic HDFs by Western blot analysis of phosphorylated Protein Kinase B (Akt) (Ser473; p-Akt), a downstream target of PI3K, and phosphorylated p38 (p-p38). In prolonged hypoxia, p-ERK levels peaked at 8 h after hypoxia exposure, whereas the total ERK protein level remained unaltered. We also found that the p-Akt level peaked 4 h after hypoxia exposure. Lastly, the hypoxia-induced phosphorylation of p38 peaked 8 h after hypoxia exposure (Figure 4). 

### 2.4. Phenotypic EMT Markers Are Expressed in HDFs under Hypoxia 

**Figure 5:**
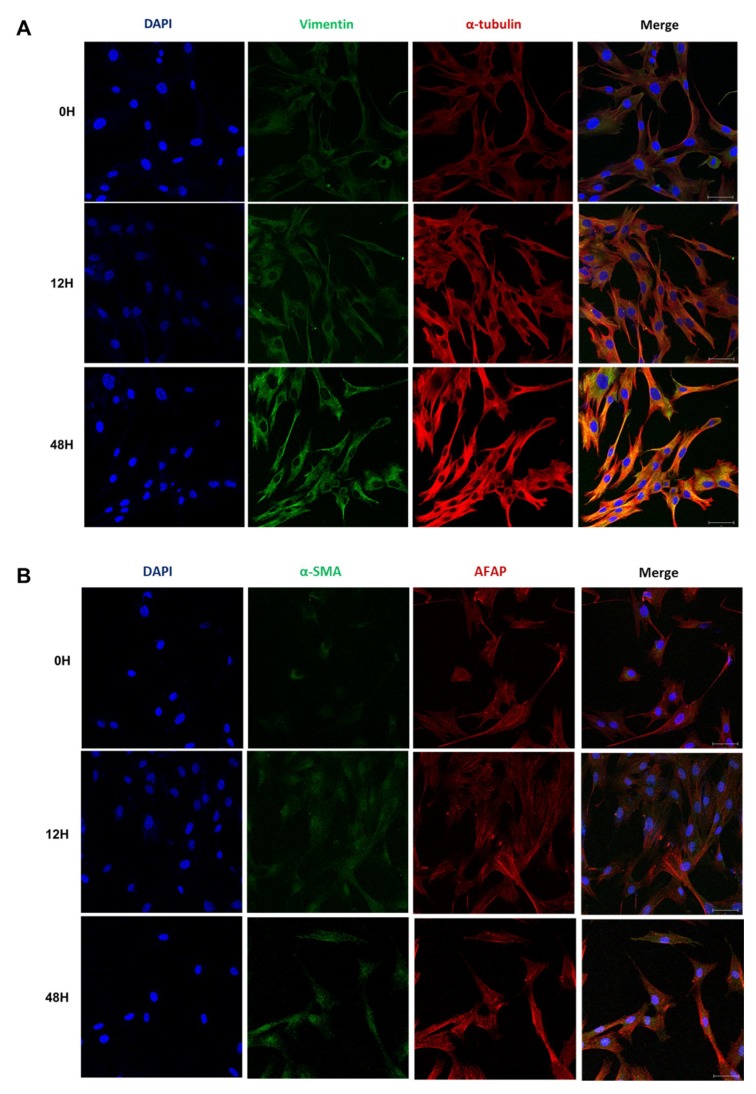
Expression of phenotypic markers of EMT under hypoxia. (**A**) Immunofluorescence analysis of vimentin and α-tubulin in HDFs under hypoxia for 12 h. (**B**) Immunofluorescence analysis of alpha-smooth muscle actin (α-SMA) and actin filament-associated protein 1 (AFAP) under hypoxia for 12 h; DAPI (blue), vimentin (green, upper), α-SMA (green, lower), α-tubulin (red, upper), AFAP (red, lower). Results are representative of three independent experiments, scale bar = 50 µM.

Hypoxia can induce phenotypic changes in HDFs that are mediated by HIF-1α and TGF-β signaling. We cultured HDFs under hypoxia for 12 h and evaluated the expression of phenotypic markers of EMT related to ECM accumulation. We found that the expression of vimentin, which is associated with the invasive phenotype of fibroblasts [22], as well as α-tubulin showed a gradual increase for up to 48 h after hypoxia exposure (Figure 5a). The expression of cytoskeletal proteins that contribute to EMT, including alpha-smooth muscle actin (α-SMA) [9] and actin filament-associated protein 1 (AFAP), were also increased by hypoxia (Figure 5b). The expression of α-SMA in fibroblast stress fibers contributes to their increased contractile ability, and is critical to tissue remodeling [23,24]. Additionally, as the exposure to hypoxia increases, we observed cortical organization of AFAP representing the increase of stress fibers in HDF [25]. The increased expression of AFAP can be suggestive of phenotypic change of HDF to a myofibroblast phenotype in response to hypoxia [24]. 

### 2.5. Hypoxia Induces a Phenotypic Switch of Fibroblasts to Myofibroblasts 

**Figure 6:**
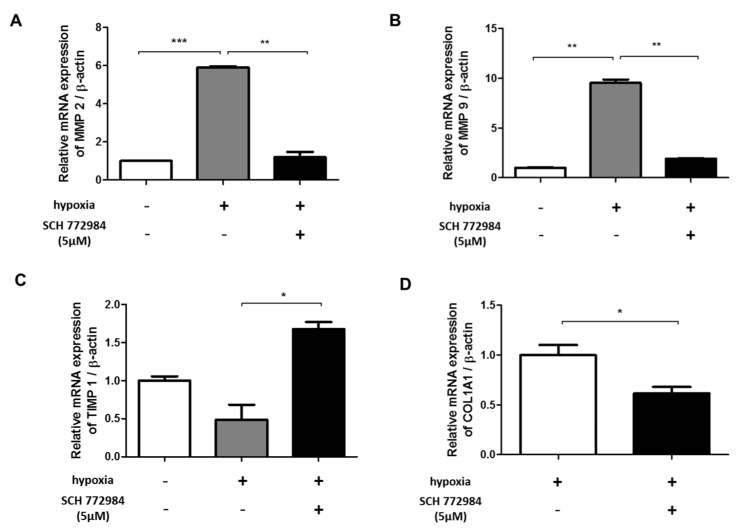
Effect of an ERK-specific inhibitor (SCH 772984) on MMP/TIMP and collagen levels in hypoxic HDFs. Effect of ERK inhibitor treatment on (**A**) MMP-2, (**B**) MMP-9, (**C**) TIMP-1, and (**D**) type 1 collagen levels in HDFs cultured with (+) or without (-) under 72 hour-hypoxia exposure or SCH 772984 treatment. Data are shown as mean ± SD. * *p* < 0.05, ** *p* < 0.01, *** *p* < 0.001.

HIF-1α regulates the expression of genes involved in ECM remodeling, including matrix metalloproteinases (MMPs) and tissue inhibitors of metalloproteinases (TIMPs) [26]. During the granulation phase of wound healing, activated fibroblasts acquire a myofibroblast phenotype associated with increased α-SMA expression [23]. In cutaneous wound healing, the phenotypic switch of fibroblasts to myofibroblasts is mediated by the MMP pathway [6]. The hypoxia-induced phenotypic switch of fibroblasts to myofibroblasts through MMPs/TIMPs is observed in systemic organ fibrosis [26,27]. We examined MMP-9 and MMP-2 production in hypoxic and normoxic fibroblasts. qRT-PCR analysis showed significant increases in MMP-2 (Figure 6a) and MMP-9 (Figure 6b) expression in HDFs exposed to hypoxia. The activation of MMPs is regulated by TIMPs, with TIMP-1 being a natural inhibitor of MMP-9. In keratinocytes, hypoxia increases cell motility via changes in MMP-9/TIMP-1 activity [26]. We found decreased expression of TIMP-1 in hypoxic fibroblasts compared with normoxic controls (Figure 6c). These results indicate that hypoxia induces changes in the expression of MMP-9 and TIMP-1 that contribute to collagen remodeling. 

### 2.6. ERK Inhibition Reduces Hypoxia-Induced ECM Deposition

ERK signaling controls cellular processes associated with fibrosis and myofibroblast transformation [28]. Recently developed inhibitors of the ERK pathway can reduce tumor invasion and organ fibrosis [29]. SCH772984 is a potent inhibitor that selectively prevents ERK1/2 phosphorylation. Unlike earlier MAPK/ERK-targeting agents, SCH772984 does not bind or inhibit MEK phosphorylation, and thus selectively targets ERK1/2 phosphorylation [30]. We found that the hypoxia-induced changes in MMP-2,9 and TIMP-1 levels were inhibited by SCH772984 (Figure 6a–c). Additionally, SCH772984 reduced the transcriptional level of type I collagen by 38.3% in hypoxic HDFs (Figure 6d).

**Figure 7:**
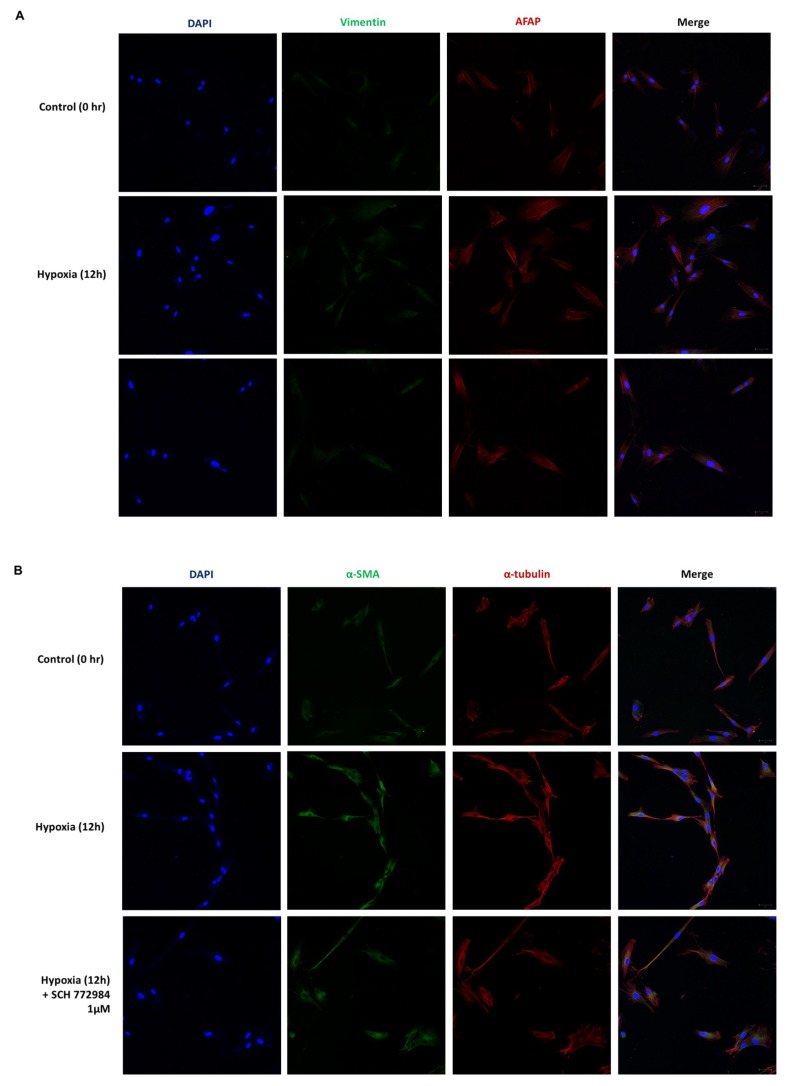
Effect of an ERK-specific inhibitor (SCH 772984) on phenotypic markers of EMT under hypoxia; DAPI (blue), vimentin (green, upper), α-SMA (green, lower), AFAP (red, upper), α-tubulin (red, lower). (**A**) Increased expression of AFAP under hypoxia for 12 h were reduced after the treatment of ERK inhibitors. (**B**) Increased expression of α-tubulin under hypoxia for 12 h were reduced after the treatment of ERK inhibitors. Results are representative of three independent experiments, scale bar = 50 µM.

To further demonstrate that hypoxia-induced ERK phosphorylation mediates ECM remodeling, we analyzed the expression of phenotypical markers of EMT in hypoxia-exposed HDFs using an ERK inhibitor. The hypoxia-induced increase in AFAP and α-SMA were reduced after treatment with an ERK inhibitor (Figure 7). 

## 3. Discussion

We examined the downstream molecular mechanisms of cutaneous fibrosis associated with wound healing by performing an in vitro assay of fibroblasts exposed to hypoxia. Our results suggest that phosphorylated ERK is a key mediator in the hypoxia-induced EMT of fibroblasts during wound healing. Hypoxia-induced ERK signaling was associated with increased levels of EMT markers, TGF-β, CTGF, and type 1 collagen in HDFs. Thus, ERK phosphorylation is a potential therapeutic target for cutaneous fibrosis. 

Keloid pathogenesis is associated with abnormal fibroblast activity and tissue fibrosis [31] that involves enhanced fibroblast migration and invasion [32]. Hypoxia stimulates fibroblasts to undergo proliferation and differentiation, resulting in ECM deposition [11,17]. HIF-1α is a critical mediator of the EMT process and induces TGF-β and CTGF expression [20]. TGF-β contributes to skin fibrosis during abnormal cutaneous wound healing [9,33], and CTGF mediates myofibroblast differentiation and disease progression in kidney and lung fibrosis [34,35]. Moreover, previous reports show increased HIF-1α and CTGF expression in various conditions involving skin fibrosis [9,20]. In the present study, we confirmed that hypoxia increases TGF-β and CTGF expression in HDFs. Therefore, our observations show that hypoxia can induce phenotypic markers of EMT related to ECM accumulation. 

MAPK/ERK signaling is involved in both normal wound healing and pathogenic fibrosis. Upon tissue injury, ERK phosphorylation participates in the transcriptional response to hypoxia, and its mediators directly contribute to HIF-1α activation. In mesenchymal cells, hypoxia triggers cell proliferation and the activation of ERK1/2 and AKT [36,37]. We also observed that KFs and keloid tissue exhibit increased levels of p-ERK, and that hypoxia increases AKT and p38 phosphorylation in HDFs. There are several reports indicating the PI3K/AKT pathway induces HIF-1α transcription, stabilization, and recruitment of coactivators [38]. Additionally, p38 MAPK is activated by oxidative stress, and is involved in cell differentiation, survival, and apoptosis [39]. In tumor models, endogenous p38 MAPK activity is correlated with cancer cell invasiveness [40]. AmoI g various mediators of cell signaling triggered by hypoxia, direct phosphorylation of ERK 1/2 is known to affect the nuclear localization and transcriptional activity of HIF-1α [38].

We observed acute activation of ERK signaling gradually increasing within 30 min of exposure to hypoxia, which was in concordance with the results of studies of cells from different origins or diseases [13,41]. In vitro tumor models show that p-ERK is required for TGF-β-induced EMT, and exogenous TGF-β can cause ERK phosphorylation upon SMAD3 phosphorylation [42,43]. In prolonged exposure to hypoxia, we observed sustained ERK phosphorylation up to 8 h. Additionally, the activation of AKT and p38 peaked at 4 and 8 h, respectively, after exposure to hypoxia. AKT plays a major role in hypoxia-induced HIF-1α activation, yet its regulation is cell-type dependent [44]. In the wound healing process and associated fibrosis, we observed a similar upregulation pattern of p-AKT [45]. Further studies using AKT inhibitors will be necessary to determine the downstream effects of AKT in response to hypoxia-induced tissue fibrosis. Therefore, we speculate that prolonged exposure to a hypoxic microenvironment triggers sequential activation of fibrosis mediators via p-ERK-triggered HIF-1α activation in cutaneous fibrosis. 

To confirm the role of ERK phosphorylation in ECM deposition and skin fibrosis, we treated hypoxic HDFs with a selective inhibitor of ERK1/2, SCH772984. MEK/ERK signaling is a promising therapeutic target for cancer stemness and cutaneous fibrosis [27,46]. We found that selective inhibition of ERK phosphorylation attenuated collagen production and ECM remodeling triggered by HIF-1α, which was supported by the assessment of MMP and TIMP expression profiles. Moreover, inhibition of ERK phosphorylation resulted in reduced expression of phenotypic markers of EMT. Therefore, ERK inhibitors could potentially be clinically applied to treat abnormal fibrosis, and further research is needed to identify safe candidates.

In conclusion, our results suggest that hypoxia triggers the transition of fibroblasts into myofibroblasts during cutaneous wound healing. We found that ERK phosphorylation is a critical mediator of hypoxia-mediated abnormal wound healing, and we provide evidence that selective inhibition of ERK signaling ameliorates hypoxia-induced fibrogenesis of HDFs. Additional understanding of the role of ERK signaling in skin fibrosis could help lead to a new clinical approach to treating and preventing abnormal wound healing. 

## 4. Materials and Methods

### 4.1. Cell Culture and Keloid-Derived Fibroblasts

Keloid tissue was collected from three patients with active-stage keloids after obtaining informed consent in accordance with the Declaration of Helsinki. Normal HDFs and KFs were derived from patient tissue and purchased from the American Type Culture Collection (Manassas, VA, USA). After separation, cells were cultured in Dulbecco’s modified Eagle’s medium (Gibco, Grand Island, NY, USA) to which 10% heat-inactivated fetal bovine serum, actinomycin, penicillin (30 U/mL), and streptomycin (300 μg/mL) were added. We replaced the culture medium every 2–3 days. Hypoxia exposure was conducted using Hypoxystation H35 (Don Whitley Scientific, Shipley, UK) with 1% O_2_, 5% CO_2_ and 94% N_2_ at 37 °C at specific time points. All experiments were approved (16 May 2017) by the Yonsei University College of Medicine Institutional Review Board (IRB No. 4-2017-0259).

### 4.2. Real-Time PCR Analysis 

The TRIzol reagent (TaKaRa Sake, Berkley, CA, USA) was used to extract total RNA from cultured cells according to the manufacturer’s instructions. cDNA was synthesized using a Maxime RT premix kit (iNtRON Biotechnology, Seongnam, Korea). Real-time PCR was performed with SYBR Green PCR Master Mix or TaqMan PCR Master Mix (Applied Biosystems, Foster City, CA, USA) using an ABI detection system (Applied Biosystems, Foster City, CA, USA). Relative differences in gene expression were calculated (∆∆Ct) and reported as fold induction (2^−∆∆Ct^). PCR primers are provided in Table 1.

### 4.3. Western Blotting Analysis

Cells were harvested and lysed in RIPA lysis buffer (Biosesang, Seongnam, Korea) containing a protease inhibitor (PPI 1015, Quartett, Berlin, Germany), and protein concentrations were determined using a BCA protein assay (Sigma-Aldrich, St. Louis, MO, USA). Protein (20 µg) was fractionated by SDS-PAGE and transferred to nitrocellulose membranes. Membranes were immunoblotted with specific primary antibodies (listed in 4.6. Antibodies and reagents) overnight at 37 °C, followed by secondary antibodies conjugated to horseradish peroxidase. Immunoblots were developed with ECL reagent (Ab Frontier, Seoul, Korea) and monitored by a luminescence image analyzer (LAS-4000 Mini, Fujifilm Life Sciences, Tokyo, Japan). The optical densities of the bands on the developed film were analyzed using ImageJ software (National Institutes of Health, Bethesda, MD, USA, https://imagej.nih.gov/ij/). Protein levels were normalized to those of actin or to the ratio of phosphorylated and total isoforms. Relative quantitation is expressed as fold-induction compared to control conditions.

### 4.4. Cell Viability Analysis

Normal fibroblasts and KFs were seeded in triplicate at 5000 cells per well in 96-well plates. After overnight incubation under normoxic or hypoxic conditions, the ERK inhibitor SCH772984 was added to each well at the indicated concentration. Cell viability was measured using a cell counting kit (CCK-8; Dojindo, Kumamoto, Japan). At specific time points, 10 μL CCK-8 reagent was added to the medium in each well, and plates were incubated for 4 h at 37 °C. Absorbance was measured at 450 nm using a microplate reader (Molecular Devices, Sunnyvale, CA, USA).

### 4.5. Immunohistochemistry

Immunohistochemistry was performed on formalin-fixed, paraffin-embedded skin tissue sections (4 µm thick). Sections were boiled in 1× citrate buffer (Sigma-Aldrich) for 10 min for antigen unmasking. Slides were incubated with 3% H_2_O_2_ on ice for 10 min, followed by a blocking step using 5% bovine serum albumin. After washing with phosphate-buffered saline (PBS), a p-ERK primary antibody (1:400) was applied to deparaffinized slides at 4 °C overnight. A DAKO peroxidase/(3,3-diaminobenzidin) DAB detection kit (DAKO, Carpinteria, CA, USA) was used for detection. Slides were stained with hematoxylin to visualize nuclei.

### 4.6. Antibodies and Reagents

p-p38 (9215), p38 (9212), p-ERK (4370), ERK (9102), p-AKT(S473) (4060), and AKT (4691) antibodies were purchased from Cell Signaling Technology (Danvers, MA, USA). α-SMA (ab7817), collagen 1 (ab34710), HIF-1α (ab179483 for western blot, ab1 for immunofluorescence), alpha tubulin (ab18251), vimentin (ab 8978), goat anti-mouse IgG H&L (HRP, ab6789), and goat anti-rabbit IgG H&L (HRP, ab6721) antibodies were purchased from Abcam (Cambridge, UK). AFAP (NBP1-90216) antibody was purchased from Novus (Littleton, CO, USA). Alexa Fluor 488 goat anti-mouse IgG and Alexa Fluor 555 goat anti-rabbit IgG antibodies were purchased from Invitrogen (Carlsbad, CA, USA). Beta-actin (sc-47778) was purchased from Santa Cruz Biotechnology (Dallas, TX, USA). SCH772984 was purchased from Selleckchem (Houston, TX, USA) and stored in 5 mM dimethyl sulfoxide (DMSO) at −20 °C. 

### 4.7. Confocal Imaging

Normal fibroblasts were seeded in a 4-well culture slide (SPL, Pocheon, Korea) for 24 h, fixed with 4% paraformaldehyde in PBS for 10 min, permeabilized with 0.2% Triton X-100 in PBS for 5 min at room temperature, and incubated with blocking solution (0.1% PBS-Tween 20 containing 1% bovine serum albumin) for 30 min. Primary antibodies (1:500) were applied for 24 h at 4 °C. After washing, slides were incubated with secondary antibodies (1:1000) for 2 h at room temperature in the dark, rinsed, and counterstained with DAPI (Vector Laboratories Inc., Burlingame, CA, USA). All fluorescence images were observed under a Zeiss Confocal LSM 700 microscope (Zeiss, Oberkochen, Germany). 

### 4.8. Statistical Analysis

Data are shown as mean ± standard error of the mean (SEM) and were analyzed using paired *t*-tests or one-way analysis of variance, with *p* < 0.05 considered statistically significant. SPSS version 23.0 (SPSS Inc., Chicago, IL, USA) was used for all statistical analyses.

## Figures and Tables

**Table 1:** Primer lists.

Target Gene	Primer Sequences (5′–3′) or Assay ID	Reference
E-cadherin	Forward: CACCACGGGCTTGGATTTTG Reverse: TGGGGGCTTCATTCACATCC	[47]
N-cadherin	Forward: TCAGGCGTCTGTAGAGGCTT Reverse: ATGCACATCCTTCGATAAGACTG	[48]
Vimentin	Forward: GACGCCATCAACACCGAGTT Reverse: CTTTGTCGTTGGTTAGCTGGT	[49]
TGF-beta	Forward: ACCCACAACGAAATCTATGACA Reverse: GCTGAGGTATCGCCAGGAAT	[50]
HIF1A	Forward: ACTCATCCATGTGACCACG Reverse: TAGTTCTCCCCCGGCTAG	[51]
COL1A1	Forward: AAGGTGTTGTGCGATGACG Reverse: TGGTCGGTGGGTGACTCTG	[50]
Beta-actin	Forward: CTACCTCATGAAGATCCTCACCGA Reverse: TTCTCCTTAATGTCACGCACGATT	[52]
CTGF	Hs00170014_m1	
MMP9	Hs00957562_m1	
MMP2	Hs01548727_m1	
TIMP2	HS00234278_m1	
TIMP1	Hs01092512_g1	
COL1A1	Hs00164004_m1	
Beta-actin	Hs01060665_g1	

## Abbreviations

AFAP	Actin filament associated protein 1
CTGF	Connective tissue growth factor
ECM	Extracellular matrix
EMT	Epithelial-mesenchymal transition
ERK	Extracellular signal-regulated kinase
HDF	Human dermal fibroblast
HIF-1α	Hypoxia-inducible factor-1α
KF	Keloid fibroblast
MAPK	Mitogen-activated protein kinase
MMP	Matrix metalloproteinase
PBS	Phosphate-buffered saline
PI3K	Phosphatidylinositol-4,5-bisphosphate 3-kinase
TGF-β	Transforming growth factor-β
TIMP	Tissue inhibitor of metalloproteinases
